# Phenomenological reflections on grief during the COVID-19 pandemic

**DOI:** 10.1007/s11097-022-09840-8

**Published:** 2022-07-28

**Authors:** Matthew Ratcliffe

**Affiliations:** Department of Philosophy, University of York, YO10 5DD Heslington York, UK

## Abstract

This paper addresses how and why social restrictions imposed during the COVID-19 pandemic have affected people’s experiences of grief. To do so, I adopt a broadly phenomenological approach, one that emphasizes how our experiences, thoughts, and activities are shaped by relations with other people. Drawing on first-person accounts of grief during the pandemic, I identify two principal (and overlapping) themes: (a) deprivation and disruption of interpersonal processes that play important roles in comprehending and adapting to bereavement; (b) disturbance of an experiential world in the context of which loss is more usually recognized and negotiated. The combination, I suggest, can amount to a sort of “grief within grief”, involving a sense of stasis consistent with clinical descriptions of prolonged grief disorder.

## Introduction

This paper investigates how and why people’s experiences of grief were affected by social restrictions imposed during the COVID-19 pandemic. I am concerned primarily with “grief” in a narrow sense of the term: an emotional response to *bereavement*. However, this also requires a consideration of wider experiences of loss that are sometimes referred to as grief, which—as we will see—can contribute to how we experience grief over the death of a person.^1^ There is no simple answer to the question of how grief has been and continues to be affected by social restrictions of one or another kind. Restrictions during the pandemic affected people in very different ways at different times, depending on a range of factors. While some lost long-term businesses or careers as their personal lives also fell into disarray, others enjoyed baking sourdough bread and playing games in the garden rather than travelling to work. Experiences of grief are also highly variable, depending—in part—on one’s biography and personality, the nature of the relationship, the circumstances of the death, and one’s current situation. Hence, my aim in this paper is not to provide a set of generalizations concerning how grief is affected by measures of types *x, y*, and *z*. Instead, I want to sketch a way of conceptualizing the effects of pandemic restrictions on people’s grief, as experienced at particular times and also over time. As well as illuminating the nature of the experiences themselves, this helps us to appreciate *why* grief has been affected in these ways. Granted, phenomenological analyses are not concerned primarily with causal explanations of experience. Nevertheless, by better understanding how experiences of grief are structured, we also come to see why they are susceptible to certain changes under certain conditions. Conversely, a consideration of those changes can tell us something about the phenomenological structure of grief more generally. So, there is a relationship of mutual illumination: we come to better understand the effects of social restrictions by attending to the phenomenology of grief and vice versa.

In what follows, I will emphasize how grief involves comprehending and negotiating a profound and pervasive phenomenological disturbance over time, in ways that depend on interpersonal relations and participation in a shared social world. With the reduction, alteration, or absence of various kinds of interpersonal and social interactions, what is lost is a form of “scaffolding” that more usually regulates experience, thought, and activity over time and assists us in negotiating upheaval.^2^ This scaffolding plays a number of distinct roles. For instance, to the extent that one is able to continue interacting with a shared social world, one retains access to norms for interpreting experience and participating in shared activities. Social activities also contribute to the sustenance and revision of a life structure. In addition, interactions with particular individuals can help one to make sense of what happened and what is now the case.

It has been suggested that more extreme measures such as national lockdowns affected people’s grief in ways that increase the likelihood of pathological grief developing. In light of this, I will begin by noting the relevance to this discussion of proposed distinctions between typical grief and what is increasingly referred to as *prolonged grief disorder*. I will then turn to my two main themes, both of which relate to that distinction. First of all, grief involves making sense of things in ways that frequently depend on interpersonal interactions. Second, grief involves recognizing, comprehending, and navigating a disturbance of one’s experiential world over time. I will show how disruption of the two can result in a sense of *stasis*, enveloping both one’s grief and the temporal structure of one’s life. One responds to the death against the backdrop of an experiential world that is already permeated by loss. Furthermore, one lacks access to interpersonal processes that facilitate engagement with loss. With this, there is a “grief within grief”, which inhibits the ability to accommodate what has happened.

## Pathological grief

A growing literature suggests that especially intense, prolonged, and arguably pathological experiences of grief are becoming more prevalent in the wake of the COVID-19 pandemic (e.g., Gesi et al., 2020; Goveas & Shear, 2020; Selman, 2021). To understand why that might be, we first need to identify the distinguishing characteristics of pathological grief. In recent years, various different terms have been proposed for referring to pathological grief, including “complicated grief”, “traumatic grief”, and “persistent complex bereavement disorder”, accompanied by substantially overlapping diagnostic criteria. A consensus now seems to have formed around the diagnostic category “prolonged grief disorder”, which was accepted for inclusion in the World Health Organization’s ICD-11 and, subsequently, the 2022 text revision of the American Psychiatric Association’s DSM-5.^3^ Diagnostic criteria in the two manuals are largely complementary, although not identical. According to DSM-5-TR, prolonged grief disorder is characterized by an intense yearning for or preoccupation with the deceased. This is accompanied by at least three of eight other symptoms from a list including disturbance of identity, disbelief, avoidance of reminders, types of emotional pain, problems reintegrating into the social world, emotional numbness, a sense that life lacks meaning, and loneliness. Prolonged grief is also said to involve impaired social or occupational function, to be inconsistent with culturally expected responses, and to be inexplicable in terms of other established categories of mental disorder (Prigerson et al., 2021, p.84). ICD-11 identifies much the same symptoms, but includes a minimum duration of six months, while DSM-5 proposes twelve months.

After so many years of debate, the decision to approve prolonged grief disorder for inclusion in the DSM may well have been hastened by the COVID-19 pandemic. It has been widely suggested that the prevalence of prolonged grief disorder increased during the pandemic and is set to increase still further (Eisma et al., 2020; Selman, 2021). Hence, the need for agreed and effective diagnostic categories has become more pressing. Experiences of being unable to grieve “properly” have also been very much in the public eye throughout the pandemic. There have been frequent media reports of grief experiences that are especially intense, painful, and conflicted, which do not become more manageable with time. Article titles include the likes of “Processing grief during a pandemic, when nothing is normal”; “The pandemic will pass. Our grief will endure”, and “How coronavirus has transformed the grieving process.”^4^ In addition, bereavement charities have sought to raise awareness of the effects of the pandemic and associated social restrictions on how people grieve.^5^

In what follows, I do not take a stance on whether certain forms of grief are indeed pathological or which criteria ought to be invoked to make such distinctions. Rather, my aim is to better understand why social restrictions of various kinds might lead to forms of experience that are consistent with clinical descriptions of prolonged grief disorder. This, I suggest, requires phenomenological work. To see why grief is susceptible to certain changes under certain conditions, we need to understand how experiences of grief are structured, at a given time and also over time. Above all else, it is important to acknowledge how the course of grief is shaped and regulated by relations and interactions with other people. This is especially so when contemplating the frozen, unchanging grief that some have reported experiencing during the pandemic. For instance, one newspaper article describes how social restrictions made it difficult to “process” the loss of the author’s mother, leading to “a freezing of the grieving process”.^6^ More specific concerns have been raised about the increased likelihood of prolonged grief in response to COVID-19 deaths, which can be especially distressing (Goveas & Shear, 2020; Pearce et al., 2021). In addressing the impact of social restrictions upon people’s experiences of grief, my concern is with bereavements more generally. Nevertheless, it is important to acknowledge that additional factors will be involved in many cases, as when deaths are exceptionally traumatic regardless of the effects of social restrictions.

A consistent theme in discussions of pandemic grief is the impact of being isolated from other people. However, there are several different aspects to this. One may have been unable to spend time with a person during the days, weeks, months, or even years leading up to their death and at the time of the death. Following the death, deprivation of social contact can encompass many different situations and activities, which are important in different ways: formal events; mundane social interactions; spending time with family and friends.^7^ How and why might grief be affected by these various privations? To answer that question, I will first sketch an account of what I take to be—for current purposes, at least— the most relevant aspects of the phenomenology of grief. Following this, I will reflect on some first-person accounts of grief during the pandemic.

## The personal and world-oriented dimensions of grief

Grief is plausibly construed as a dynamic, temporally extended process, rather than an enduring state, episode, or disparate sequence of episodes (Goldie, 2012; Ratcliffe, 2017; 2022). In recent work, I have suggested that there are two principal aspects to this process, which are inextricable and together span the full diversity of grief experiences. First of all, grief involves comprehending and navigating a—sometimes profound—disturbance of one’s experiential world. Second, it involves making sense of what has happened to a person and to one’s relationship with that person. Importantly, both are reliant upon relations and interactions with other people, against the backdrop of a shared social world (Ratcliffe, 2022). 

How might grief be said to affect one’s *world*? In mundane, everyday situations, how we experience our surroundings involves taking things to *matter* in a range of familiar ways, which retain a degree of consistency over time. Certain things appear immediately salient and significant to us while others do not, in ways that do not generally require explicit inferences from experience. These things are not experienced as significant in an atomistic fashion; how one thing matters depends on how it relates to various other things that also matter. For instance, when I head downstairs to make my lunch in a few minutes’ time, I will encounter the fridge, kettle, bread bin, and cutlery drawer as interrelated in familiar ways that reflect organized patterns of activity. This experience of a cohesive, practically meaningful scene does not depend simply on whatever concerns I might have at the relevant time; it further depends on my having various longer-term projects, habits, pastimes, and expectations, relative to which things matter to me in the ways they do. For example, how I currently experience my office reflects the project of writing an article on pandemic grief, which is part of a larger, collaborative project on the phenomenology of grief, which presupposes my employment as an academic philosopher, something that comes with a host of other projects, commitments, and habitual expectations. All of this brings structure to my experiences and activities, by determining the kinds of significant possibilities that things offer, eliciting patterns of practically oriented thought, and constraining the scope of situationally relevant activities.

In a broad sense of the term “value”, we might say that the significance with which things are imbued, and which one’s thoughts and activities also take for granted, reflects a largely stable network of values that together distinguish one as a person. The sustenance of this structure can come to depend upon one’s relationship with another person in numerous ways. Consequently, bereavement has the potential to throw one’s experiential world into disarray. Projects may only make sense insofar as one has enduring commitments to that person and strives to do various things for them or with them. Furthermore, pastimes may be what *we* do together, while the fulfilment of a host of habitual expectations depends on that person’s actions. Thus, another person can be more than just a cherished and irreplaceable entity within one’s world. They can also become a condition of *intelligibility* for a world that is ordinarily presupposed as a backdrop to one’s experiences, thoughts, and activities, including one’s interactions with the person in question (Ratcliffe, 2017; 2022).

Hence, in the event of a death, we are sometimes faced with the occurrence of something that undermines the very world within which it is experienced as occurring. This tension can envelop a wide range of experiences, thoughts, and activities over a lengthy period of time. So, we might grasp the truth of the proposition “that person is dead”, but at the same time find it somehow impossible. We recognize its undeniable truth while encountering a world still at odds with it, a world that continues to imply the person’s potential presence in all manner of way. As Juliet Rosenfeld writes, in an account of the grief she experienced following her husband’s death:

In my external world, I lived in the strangeness of a house, his house, our house, where he was not, yet where everything that was his was still in place. The impossibility of that, I think now. His toothbrush and socks next to mine. (Rosenfeld, 2020, p.248)

Such tensions are not resolved in a smooth, gradual way; at certain times, they are especially pronounced. During the course of grief, there might be numerous experiences of presence, absence, anticipation, and negated expectation, which vary in their duration and degree of localization. They can be construed as integral to a longer-term process whereby the structure of one’s world eventually becomes reconciled (although perhaps not entirely) with the fact of the death (Attig, 2011; Ratcliffe, 2016; 2022; Fuchs, 2018; Read, 2018). The alteration of an unsustainable experiential world is not something that happens instantly or passively. It involves interacting with one’s surroundings over time, in ways that facilitate the revision of habitual patterns of experience, thought, and activity.

It is not simply that one structured experiential world eventually comes to be replaced by another or, at least, revised. It is also important to recognize the space in between pre- and post-bereavement worlds. Navigating a dynamic, temporally extended disturbance of one’s world can involve different balances between retention, loss, and revision of life structure. Structure must be lost in order for new structure to be established. However, losing too much at once gives rise to profound, pervasive, and unsettling experiences of indeterminacy and lack of direction. Without the network of projects, commitments, habits, and expectations that previously shaped experiences, thoughts, and activities, there is nothing to specify what is to be done in a given situation. It is not merely that one does not *know* what to do; norms that previously guided thoughts and activities are gone and so there is no fact of the matter. With a loss of life structure and nothing yet to replace it, one is thus left disorientated, lost (Ratcliffe, 2022, Chap. 4).

In reflecting on the phenomenology of grief, we should not over-emphasize the impact of bereavement upon one’s own world or life structure; grief is not merely concerned with *what has happened to me*. Another important aspect of grief concerns how we experience, think about, and relate to the person who has died, and how we make sense of what has happened to them. Amongst other things, this involves the reorganization of autobiographical memories (Goldie, 2012). Due to the person’s death, and sometimes the manner in which they died, the significance of earlier memories is altered; they no longer relate to one’s present or to one’s anticipated future in the ways they once did. Consequently, there can be contrasting and even conflicting perspectives upon past events, which interact with one another and change over time. This reorganization does not just involve altering one’s biography to take account of the unequivocal *absence* of a person from one’s present and future. It can also contribute to or interfere with the formation of what have been termed “continuing bonds” with the deceased. Rather than “letting go” of the deceased, a substantial and diverse body of findings suggests that we tend instead to sustain relationships in various ways. They are not simply retained unaltered, as though the death never occurred. Instead, the bereaved are tasked with establishing a form of enduring connection that is consistent with the person having died (Klass, 1999, 2006; Klass, Silverman, and Nickman, eds, 1996; Klass and Steffen, eds, 2018).^8^

A grief process does not unfold in a predictable manner that can be attributed wholly to an individual’s internal psychology. As we will see in turning to first-person accounts of grief during the pandemic, its temporal structure is fragile and depends in important ways on interactions with other people and with shared social environments. Some such interactions might be said to *regulate* the course of grief over time. “Emotion regulation” is sometimes conceived of primarily in terms of internal psychological processes that influence the initiation, course, and conclusion of brief emotional episodes (e.g., Gross 1999; 2001; 2014). However, it has been suggested that emotion regulation in humans is reliant to a considerable extent on processes that are interpersonal in nature, from patterns of interaction in early attachment to relationships between adults (e.g., Thompson 1994; Mikulincer et al., 2003; Campos et al., 2011; Kappas, 2011; Varga & Krueger, 2013). Some such processes also depend upon wider social and cultural arrangements (e.g., Mesquita et al., 2014). In considering temporally extended, dynamic, multi-faceted processes such as grief, it is especially apparent that much of the regulatory work is interpersonally and socially distributed (Ratcliffe & Byrne, 2022).

Emotion regulation has been defined specifically as the direct or indirect regulation *of* emotion, in contrast to the regulation of something else *by* emotion (Gross, 1999, 2001). But, in the case of grief, the two cannot be cleanly distinguished. What we have is a multi-faceted, temporally extended process whereby emotional experiences are shaped and reshaped through interactions with a social environment in ways that then influences those interactions, and so forth. Given this, one might be tempted to restrict the term “emotion regulation” to more clearly delineated, short-term episodes involving heightened bodily arousal. However, it is arguable that this would exclude *exactly* those emotional experiences that affect us most profoundly and play the most important roles in our lives—protracted upheavals that involve comprehending and navigating pronounced and wide-ranging disturbances of one’s world. That said, little rests on such terminological choices and we could just as well refer to the interpersonal and social dimensions of “coping”, rather than emotion regulation (Dunahoo et al., 1998). Either way, a point to keep in mind when reflecting on experiences of grief during the pandemic is that grief and its course over time depend in various ways on relations and interactions with others.

There is a need be more specific about the various different contributions made by other people and shared social environments to the experience of grief and its temporal structure. For instance, other people may contribute to the establishment of new life structure and to the sustenance of whatever structure remains, by supporting the continuation of activities and also opening up new possibilities. They can also act as guides, during times when one’s life is lacking in organization that previously specified what to think and do—when projects have become unsustainable and habitual expectations no longer apply. In addition, they can be important interpretive resources, helping one to make sense of what has happened and shaping an ongoing experience of connection with the deceased (Neimeyer, 1999, 2005; Walter, 1996). Furthermore, they can contribute to practical activities that aid in reorganizing one’s world, such as sorting through possessions and finding a place for cherished objects that themselves elicit various emotional responses (Gibson, 2008). On top of this are all the shared rituals and customs that enable (and sometimes impede) our ability to be with others and interact with them. They incorporate scripts, stories, and norms for making sense of things and prescribing shared activities, which can contribute to or interfere with the comprehension of what has happened and what one now faces.^9^

By conceiving of grief in this way, we are better able to understand how and why it is affected by radical social restrictions. People are deprived of regulatory resources that more usually influence the trajectory of grief over time, contributing to the comprehension and navigation of upheaval, the ongoing interpretation of events, and a changing sense of connection with the deceased. Furthermore, the experiential world that bereavement undermines has already been profoundly altered by the widespread disruption of social life.

## Making sense of things

To further investigate how social restrictions have affected people’s experiences of grief, I will draw upon testimonies collected with colleagues via two qualitative surveys. One of these was a wider-ranging study of the phenomenological effects of pandemic restrictions (Froese et al., 2021).^10^ The other was concerned specifically with the phenomenology of grief and did not make explicit reference to the pandemic. However, it was conducted in the UK between 2020 and 2021, while social restrictions were in place (Ratcliffe, 2022, Chapter 1). In both cases, respondents were invited to provide open-ended, free-text responses to a series of questions. In reflecting on some of these responses, I will first address the theme of how one interprets what has happened and what is now the case (with particular emphasis on one’s relationship with the deceased), after which I will turn to the manner in which one’s experiential world is disrupted.

Prominent in many first-person accounts is the distress caused by not being with a person during the time leading up to their death and/or not attending a funeral or, at least, a “proper” funeral. Consider the following testimonies:

I was unable to visit her in hospital, to go to her house to offer comfort, or attend the funeral due to social distancing measures. I feel utterly devastated.

We had a Zoom funeral—it was pretty rubbish, and felt more like it was done because you are meant to have funerals than as a way to actually help process grief.

My grandma died in April aged 100 and we could only watch online. It was so sad and lonely. All the family wanted to be together and hug but we couldn’t even see each other. It felt cold and unnatural.

My dear friend died (brain tumour) and I think being unable to go to her funeral (it was online, bleak, her parents alone in a crematorium in masks—dystopian) or get together with other close friends intensified the grief.

I was unable to attend the funeral of an acquaintance and found it distressing not to be able to communicate my grief and respect by attending the funeral.

A number of more specific themes can be discerned here. The funeral itself is “cold” or “unnatural”; being unable to attend the funeral is “distressing”; and privation of bereavement-related rituals and other social activities “intensifies” grief and impedes “processing”. Respondents also emphasize deprivation of bodily contact, not being able to hug or console one another: “Not being able to hug and support family members and having to see my mum stood on her own during my dad’s funeral was tough”; “It has been very difficult trying to support her through her horrible loss while not being able to visit or hug her”; “this lack of being able to console one another definitely made the grieving process harder”. The common theme, then, is that restrictions on social activities and interpersonal contact somehow added to people’s distress.^11^

However, observing that grief becomes more intense, distressing, or otherwise challenging does little to illuminate what the relevant experiences consist of or, for that matter, *why* they might be exacerbated by a privation of interpersonal and social contact. I want to draw attention to two factors, one or both of which feature in many accounts. First of all, being unable to spend time with a person before they died and to grieve with others deprives one of important interpretive and regulative resources, which might otherwise assist in making sense of and responding to what has happened, what is now the case, and what the future may hold.^12^ For instance, something people often do together is share stories. As well as interfering with the exchange of pre-formed, already fixed narratives, social restrictions obstructed the co-construction and revision of narratives: “unable to go to the funeral of someone who died….so unable to share thoughts of that person with others at the funeral”. Such activities might otherwise help to shape and reshape the significance of events, how those events relate to one another, and how they are integrated into one’s own ongoing life and relations with others. An important consideration here is how the circumstances of the death affect one’s memories of the person and, more specifically, the significance of those memories. Consider the potential impact of not being there for someone as they died or during the preceding period:

I fear that when and if this situation resolves, I will look back on that time and feel unable to cope with the way the end of my father’s life was. I hope that I can recall that it was out of my control at that time and accept that I could not change things.

In the above account, there is an explicit concern with how things will be remembered, the kind of significance that events will take on in the context of one’s ongoing life.^13^ The changing significance of memories during grief has been addressed by Peter Goldie, who draws attention to how autobiographical memory in grief often involves a tension-riddled comingling of perspectives:

When you grieve, you look back on the past, on your time together with the person you loved, knowing now what you did not know then: that the person you loved is now dead, and that you now know the manner and time of the dying. Grief is replete with the irony of memories such as these. For example, you remember the last time you saw the person you loved, not knowing, as you do now, that it was to be the last time. And this irony, through the psychological correlate of free indirect style, will infect the way you remember it. (Goldie, 2012, p.65)

Following a bereavement, one’s perspective upon various remembered events changes. One might remember the significance that those events had at the time they occurred, as well as a significance that they retained or took on afterwards. However, things now seem radically different from both, in light of the death and perhaps also how it happened. Thus, although these perspectives comingle, they do so uneasily.

The kind of significance attached to biographical events depends on what those events led to, what values we currently hold, and what we now strive towards. Events, as remembered, *point to* various significant possibilities that may or may not have been subsequently actualized: that was when we first met; that was the moment I decided we had to leave; I remember the day you were born. These memories are integrated into a larger biography of events that unfolded in ways that matter to us, leading up to the present and ahead to possibilities that are yet to be actualized. Hence, events that subsequently occur have the potential to transform the significance of much that was. In this way, Sartre (1943/1989, pp.497-9) suggests we are able to *change* the past. How events hang together and how they matter to us depend on which possibilities we currently pursue. Especially where temporal sequences of events have an explicit narrative structure or are—at least—amenable to narration, the experience of later events transforming the significance of earlier events is commonplace. The phenomenon is not limited to one’s biography. For instance, a film or novel can be ruined by a bad ending, which ripples back to transform the significance and cohesion of what preceded it—all of that turned out not to matter; none of it made any sense in the end; they went through it all for nothing. Where one’s biography is concerned, experienced events can similarly impact upon the significant past, undermining and transforming the ways in which things matter, including one’s own actions, inactions, and relationships. With this, the kinds of emotions elicited by past events and relationships are also altered, given that which emotions we experience depends on how the objects of those emotions (including remembered situations and events) matter to us.

Importantly, then, how somebody dies, how one relates to them at that time, and how one subsequently interprets those events all have the potential to reconfigure a sense of that person’s life as a whole and how it connects with one’s own life, to destabilize and alter established patterns of experience and thought. The ability to make sense of things can be affected by a lack of interpretive resources, including a lack of social interactions that involve constructing and reconstructing narratives in ways that support reinterpretation. For instance, as Higgins (2013) has observed, co-constructing narratives with others can involve a sense of spontaneity and openness. This could prevent the consolidation of a single, fixed narrative structured around—for instance—one’s own guilt or inadequacy.

In addition, collaborative interpretive practices contribute to the kinds of connections one maintains with the deceased and which emotions predominate when thinking of them (West et al., 2021). Whether or not one is able to feel close to the person who died can also depend on wider opportunities to participate in certain social environments, go to particular places, or interact with certain objects: “I have a little pot to put some of his ashes in, but, due to Covid, I’ve not been able to get this. I feel I need something to feel I am closer to him”. Privation of interpersonal and social interactions also interferes with a more general ability to make sense of and accommodate loss: “because of Covid, we have been denied the ritual and processes that are important to dealing with the loss of a loved one”; “all of our support networks were pulled”; “my usual coping mechanisms…were not there”. All of this can contribute to the trajectory of grief, which depends—in part—on how what has happened is integrated into one’s world and how one continues to think about and relate to the deceased.

Nevertheless, it is important to reiterate that those who grieved during the pandemic will have experienced social restrictions in quite different ways. For example, an event such as a funeral is not always an opportunity to be with others and share in one’s grief. For some, it is an obligatory performance that is alienating and better avoided. One survey respondent thus writes that restrictions prohibited a funeral of the kind that would have required an uncomfortable social performance:

It has been helped, in that the “performance” I would have had to maintain as the eldest son if we had had a full funeral with the social event afterwards would have been a lot of psychological pressure. As it was, being able to grieve for him in private was less stressful. Being at the funeral with only a priest, and immediate family (socially distanced) was much easier than a full-blown event would have been.^14^

Another respondent remarked on how lockdown had helped them to “process” their grief, by providing the time needed to do so. In contrast, however, others reported experiencing an alienating sense of “unreality” during socially-distanced or on-line funerals. For them, something was strangely and profoundly lacking: “it feels disconnected and unreal. I guess that it is related to the inability to be there and grieve as usual, with other people by my side”; “The funeral was limited to 10 people and was very short. It didn’t feel real”. Why might this be? To answer that question, we need to address the way in which grief involves the disturbance and reorganization of an experiential world over a period of time.

Feelings of “unreality” are associated with grief more generally, especially in its early stages. At least some such feelings can be understood in terms of recognizing the truth of the proposition “that person is dead”, while it remains utterly at odds with a world that one continues to experience, a world that still implicates the deceased throughout: those are his shoes; that’s the café we go to every morning; here’s the sofa where we watch TV in the evening; these are our friends. There is a gradual process of “sinking in”, whereby one’s world becomes reconciled to what has happened within it (Ratcliffe, Richardson, & Millar, 2022). A “Zoom funeral” or a funeral involving only a few people, all wearing masks and standing at least two meters apart, might well seem “unreal” in a not dissimilar manner. One is unable to interact with others in ways that would otherwise involve acknowledging what has happened, seeking to comprehend it and integrate it into a shared reality. Hence, the gulf between an abstract proposition and an experiential world persists, and an event that might have operated as a bridge between them instead adds to the sense of detachment. The funeral itself, like the proposition, is disconnected from a larger reality that one continues to experience. This point applies to the effects of social restrictions more generally, which prohibited people from interacting with their social surroundings in ways that involve the repeated negation of habitual expectations concerning the deceased and the consequent reorganizing of an experiential world over time:

As I was not able to go to the funeral or see my aunt’s body, I feel like I have not accepted that she is dead. This is not helped by lockdown. I haven’t been able to go to her house so I feel like she is still there.

I imagine that if I were to go round to her house or if it had happened before (a normal) Christmas when we would usually be at their house then the loss would be contextualized and might feel more real.

I still think of him as if he were alive because I’ve been in my little lockdown bubble and haven’t had to consider his loss in real terms yet, I think.

However, it is important to add that the pre-bereavement experiential world did *not* simply carry on unchanged. I will now consider how social restrictions, at least the more extreme ones, also transformed the backdrop relative to which loss would more usually be recognized, understood, and negotiated practically over time. The experiential world was not only disrupted by the bereavement; it was already profoundly disrupted at that time.

## Lost possibilities

Many people’s experiences of grief during the pandemic arose within the context of a wider sense of loss. We could think of “loss” in terms of the subtraction of something concrete that was once *had*. Thus, someone could lose a house, a job, or a bodily capacity. However, loss experiences are not merely past-directed; they are future-oriented too. Possibilities that may once have been central to the structure of one’s life and/or the lives of others whom one cares about are experienced *as* negated or unrealizable. With this, the significance of the surrounded world is altered, at least to the extent that it presupposed those possibilities. The relevant experiences need not involve the subtraction of something concrete from the world; an experience of loss could just as well concern something that never was and will never be, something that was once anticipated and is now recognized as beyond one’s reach (Harris, ed., 2018; Ratcliffe, 2022).

Not all experiences of absence or lack amount to “loss” in this sense. For instance, we might experience the absence of a sofa that has been removed from a room or the lack of people in a train carriage. In both cases, although something is phenomenologically salient as lacking or missing, it does not amount to an experience of *loss* in the way that I have in mind. What distinguishes such experiences is that they involve the negation of possibilities that were *central to the structure of a person’s life*, to the network of projects, commitments, pastimes, and habits through which their world was organized. For some, extreme social restrictions were experienced in just this way—as a privation of possibilities that were integral to their lives and/or the lives of loved ones. Given this, even among those who did not suffer bereavements during the pandemic, experiences described in terms of “loss” and also “grief” were commonplace:

I have felt a sort of grief, having to let go of how I imagined my life would be over the course of this year, at quite a significant and long-awaited time for me personally. I’m very aware that this is nothing compared to the grief of losing a loved one, and consider myself relatively lucky in this whole situation, but it does feel like some form of grieving nevertheless.

I had quite a congenial little life which has been blown away, and I mourn it.

Some survey responses describe grief or loss over *life events* that were never marked and will never be recovered—birthdays and anniversaries; a grandchild taking those first few steps; important exams; freshers’ week at university; graduation day; the kind of wedding one had planned and hoped for; the honeymoon; being with loved ones during those precious final months of life. In such cases, the sense of loss can further extend to the possibilities of others whom one cares about:

Felt grief/loss over things my daughter has missed out on. Her A levels that she worked hard for two years on, her last day at school, prom, university offer holder day, etc. Loss of time with wider family and friends.

With measures such as national lockdowns, there is also a more pervasive sense of lack and strangeness, involving the negation of widespread habitual expectations and an associated sense of absence spanning all those habits, routines, pastimes, and ways of doing things that contribute to the mundane course of a life: “I definitely think I experienced a sense of loss in the first few weeks that was similar to grief, I guess for the world as we knew it and the life I was living”; “the loss of simple, normal things, such as going out for breakfast with my husband, or going to visit my parents at their house”. What is experienced as negated, absent, or lost is not just a set of concrete entities, situations, relationships, or events—it can include the very shape and movement of human life:

Loss of places where I have been happy that may close down for ever—local pub, cinema, restaurant, concert hall. Most—not being able to cuddle my cheery grandson when he gets tired and can’t quite fall asleep, or read to my granddaughter. And grief for time passing as we get older without new experiences and time is running out.

What is it to lack “new experiences”? The claim is not literally that *nothing* happens, that one moment no longer leads into the next. What is missing is an experience of *significant* transition, involving the pursuit and realization of possibilities that are integral to the development and transformation of a life. The little things carry on from one moment to the next but nothing of consequence happens or is anticipated to happen; there is no prospect of moving forward. We might say that what is lost includes a certain aspect of temporal experience itself—time passes but is not lived. And this is not merely a matter of things being put “on hold”; there is much that cannot be recovered or moved unscathed to a later date:

I have felt a sense of loss over missed opportunities, having planned to go on holiday and that not being possible. I have felt a small sense of loss of youth, as often people say your twenties is a time of great adventure, which has been taken from me and many others. And time is not something that can be given back.

I feel a great sense of loss over things which have given me pleasure and confirmed my sense of self throughout my life. They’re absent now and may not return soon, if at all (singing in choirs, performing, rehearsing).

Grief over the future life that is no longer likely to be available. I feel I and many of my contemporaries have lost some of the best years of our lives, when we had finished or eased off long hours of work and planned to have better social lives, enjoy freedom etc.

With a pervasive experience of loss comes a sense of *indeterminacy*; one’s surroundings no longer elicit patterns of thought and activity in the ways they once did. Hence, for many people, pandemic experience was characterized by a widespread sense of what we might call “disorientation”, involving uncertainty over what is coming next and what is to be done (Fernández Velasco et al., 2021; Ratcliffe, 2021). There is the feeling of being somehow lost, bereft of habitual patterns of expectation that were once taken for granted:

Lack of ability to plan for the future, things put on long-term hold. Everything was turned upside down and everything is different. I found it very stressful and difficult to cope for the first time in my life.

I would say that this pandemic has completely undermined the certainty I had before, either in relation to my short-term projects, my health or in relation to the long-term goals, future-oriented plans. It has completely changed the way that I used to plan my life, for the worst.

With so many known unknowns and many more unknown unknowns the future feels even more unwritten than usual.

This diffuse sense of loss, uncertainty, and disorientation also contributes to how bereavement grief is experienced. What many—although by no means all—of those who suffered bereavements during the pandemic experienced was a sort of “grief within grief”. The experience of bereavement arose within a world already infused with loss: “I have been struggling with the double weirdness of widowhood and lockdown, which made everything much harder”. The world within which one encounters personal loss is not only *subsequently* transformed by that loss; it is already lacking. In fact, experiences of loss stemming from social restrictions can be so similar to the effects of bereavement that people find it hard to distinguish the two: “The world is different for everyone now; it’s difficult to tell if this is a reaction to grief or to the Covid pandemic”.

With this, there is a diminishment of the more usual contrast between the proposition “that person is dead” and an experiential world that runs counter to it for a time. Consequently, the process of reconciling one’s world with such propositions is also disrupted. In addition to this, while the reality of the situation is sinking in, one does not experience such a stark contrast between one’s own world and a world of other people that carries on regardless. The diminution or absence of this contrast is sometimes experienced in a positive way: “lockdown actually helped me not feel so different from everyone else and I was able to manage the time and relax for the first time in over a year”; “after a while, it helped that the world was so strange as I didn’t have to deal with life going on as normal all around me while I was suffering so much”. However, even if the reduction of an alienating distance between one’s own world and that of others is experienced as comforting for a time, there remain implications for the temporal structure of a grief process, which involves reconciling the two over an extended period. And for some, the sense of being removed from a habitual world and unable to find a way back to it is a salient aspect of pandemic grief: “I’ve been in a little bubble and the Covid lockdown has reinformed this and I’m not sure what normal is anymore and how I will ever reach it”.

These forms of experience are compounded by reduced interpersonal and social interactions. One is thus deprived—to varying degrees—of processes that might otherwise help to sustain certain aspects of one’s life and provide guidance when previously taken-for-granted norms and patterns of expectation are lacking. Hence, the disorientation of bereavement is experienced against a backdrop of disorientation. And, at the same time, there is a lack of access to precisely those interpersonal and social interactions that might otherwise have helped one make sense of what is happening and reorganize one’s life. Consequently, what has happened does not “sink in” over time as it otherwise might. There is nothing for it to sink into and no path to follow. How, then, might all this influence the manner in which grief is experienced over time?

## A grief without movement

By attending to the themes identified here, we can better see why social restrictions during the pandemic might be associated with something that approximates clinical descriptions of prolonged grief disorder. Although experiences of grief and of social restrictions are both diverse, I have focused on a scenario involving the following: (a) one is deprived of interpersonal and social interactions that more usually provide interpretive and practical support for making sense of what has happened to a person and reconciling one’s life structure with a current reality; (b) one’s experiential world is not only disrupted by bereavement; it is already profoundly altered. Due to combinations of (a) and (b), neither one’s world nor one’s relationship with the deceased are reorganized in the usual ways so as to take account of what has happened. Furthermore, given a prolonged and pervasive disruption of life structure, temporal transition is not experienced in terms of the actualization of cohesively organized, practically meaningful possibilities against a backdrop of life projects. To the extent that one lacks possibilities for significant development and transformation, there is just more of the same—no prospect of transcending one’s current predicament or of recontextualizing one’s loss.

With this combination of factors (and other combinations that approximate it to varying degrees), what we have is a grief lacking in movement, a grief that does not involve comprehension of what has happened or its integration into one’s life and relationships over time. This may be experienced as static, frozen, even seemingly without end:

I feel unable to let go of the grief as I feel that I am putting it on hold while we wait for this situation to end and we are all, in a sense, fighting for survival. I feel that this is preventing me from reflecting on what has happened to our family. I feel as though my father’s death was part of a world event rather than a private family matter.

Brother-in-law died suddenly and unexpectedly not due to Covid and it feels like grief was paused as it could not run the usual course of attending funeral etc… The sense of unreality still persists as have not been able to see family and be aware of the missing person.

I don’t feel I have been able to grieve properly because of lockdown. I feel like I am stuck in the grief cycle and I can’t move on.^15^

Being unable to “let go” of one’s grief or finding that it has “paused” involves the suspension of a process whereby the significance of the death changes as it is integrated into one’s life over time. The sense of “unreality” persists, as the fact of the death remains at odds with a habitual world that has not moved to accommodate it.^16^

In reflecting on such experiences, it becomes increasingly evident that grief is not simply an emotional response that unfolds predictably over time. Instead, it is a highly variable process that is shaped and regulated by interpersonal interactions, which are themselves embedded within a shared social world. Deprived of a world with which one’s loss might be reconciled over time and of processes that facilitate its integration, grief lacks movement. Hence, an intense, unchanging grief is not to be construed exclusively or even primarily in terms of processes that are internal to individual; it is also important to recognize processes that are interpersonal and social in nature. Pandemic grief thus turns out to be phenomenologically revealing with respect to the temporally extended and fragile structure of emotional experience, our dependence on other people, and our sense of temporal change. Likewise, a phenomenological perspective that emphasizes world experience and its dependence on interpersonal and social relations can aid us in conceptualizing the considerable challenges faced by those grieving during the COVID-19 pandemic and beyond.

## Footnotes

1 For discussion of grief in response to various different non-bereavement losses, see Harris, ed. (2020).

2 See Colombetti & Krueger (2015) for a more general discussion of how we rely on forms of “scaffolding” to regulate emotional experience.

3 The ICD-11 description of Prolonged grief Disorder can be found here: https://icd.who.int/browse11/l-m/en#/http://id.who.int/icd/entity/1183832314 (last accessed 13th June 2022). For an earlier description of prolonged grief disorder and a proposal for its inclusion in both ICD-11 and DSM-5, see Prigerson et al., (2009). For a detailed historical review of changing conceptions of pathological grief, as well as the full diagnostic criteria for inclusion in DSM-5-TR, see Prigerson et al., (2021).

4 The three articles mentioned can be found here (last accessed 13th June 2022): https://www.rollingstone.com/culture/culture-features/coronavirus-grief-pandemic-emotional-fatigue-981847/. https://www.washingtonpost.com/opinions/2020/04/06/pandemic-will-pass-our-grief-will-endure/?arc404=true. https://theconversation.com/how-coronavirus-has-transformed-the-grieving-process-136368.

5 See, for example, the website of the charity Cruse Bereavement Care: https://www.cruse.org.uk/get-help/coronavirus-bereavement-and-grief (last accessed 13th June 2022).

6 See: https://www.telegraph.co.uk/family/relationships/need-grieve-mum-pandemic-has-taken-away/ (last accessed last accessed 13th June 2022).

7 Another factor, which I will not discuss here, is that many people also had limited access to support services (Selman, 2021).

8 The view that there are two broad aspects of grief, one concerned with what has happened to the practically meaningful world and the other with more specifically *interpersonal* aspects of loss, is broadly compatible with the influential “dual process model” of coping with bereavement (Stroebe & Schut, 1999). However, I am not committed to the more specific position that these aspects of grief are separable to the extent that we can “oscillate” between coping with one and then the other, although certain aspects of loss can of course be more or less salient than others at certain times.

9 See, e.g., Scrutton (2018) for discussion of ways in which shared rituals shape the experience, interpretation, and course of grief.

10 The data we collected, amounting to over 1,800 responses and 574,000 words in total, is now freely available in a publicly accessible archive. See Froese et al. (2021) for details.

11 This also applies to some of those grief experiences associated with bereavements that occurred before the pandemic: “My son died before the pandemic. It has made the experience of grief more overwhelming”.

12 See also Hernández-Fernández & Meneses-Falcón (2021) for a discussion of being unable to say goodbye and how this can lead to an enduring disbelief, sense of unreality, and lack of acceptance.

13 It can be added one’s own efforts to interpret what happened and integrate it into one’s biography could be further complicated by an appreciation of how social isolation may have affected how the deceased interpreted the significance of their life and experienced its end.

14 See also West et al. (2021) for a consideration of some of the beneficial effects associated with the absence of more usual shared practices.

15 See also West et al. (2021) for discussion of grief experiences during the pandemic that lack “forward momentum”.

16 Talk of “letting go of one’s grief” is to be distinguished from letting go of one’s attachment to the person who has died, given that the subsiding of grief is compatible with the retention of a “continuing bond” (Klass, Silverman, and Nickman, eds., 1996).
